# The pace of reduction of cardiovascular mortality in Brazil (1990 to 2017) is slowing down

**DOI:** 10.1590/1516-3180.2018.1371090219

**Published:** 2019-05-08

**Authors:** Paulo Andrade Lotufo

**Affiliations:** I MD, DrPH. Full Professor, Department of Internal Medicine, Faculdade de Medicina da Universidade de São Paulo (FMUSP), São Paulo (SP), Brazil.

The risk of death due to cardiovascular diseases, coronary heart disease (CHD) and stroke in Brazil has been declining since the 1980s.^1^ Two analyses relating to CHD and stroke mortality trends have revealed that the downward trend has continued to decline over the most recent years.^2^^,^^3^ Incontrast, in the United States, there are indications of stagnation of cardiovascular death rates.^4^

Since the 2000s, the coverage of the Brazilian national mortality surveillance system has increased, and the quality of death certification has improved. This has yielded a higher proportion of accurate diagnoses. Thus, for example, there are now fewer cases of mortality due to “heart failure” (a garbage code) and more of CHD; and fewer cases of mortality due to “hypertension” (also a garbage code) and more of stroke.^5^

I hypothesized that this improvement in quality ought to alter the real trend of mortality due to CHD and stroke. To verify this possibility, I used information from the Global Burden of Disease study, which corrects these two phenomena (i.e. increased coverage plus improved quality) through new data.^6^ To analyze differences in trends from 1990 to 2017, the annual percentage change (APC) was calculated by applying the Joinpoint regression software.^7^

Figure 1 shows that the reductions in age-adjusted mortality rates for men (A) and women (B) have been declining over the course of this period, which comprises almost three decades. However, visually, it is not possible to conclude whether the rates have flattened or have continued to fall over the last five years (2013-17). Table 1 shows the APCs according to sex and type of disease. For all cardiovascular diseases, the decline has been maintained for men, but not for women. Indeed, the flattening of the cardiovascular disease rate trend is due to the trend observed for stroke mortality, but not for CHD. The reductions in deaths due to CHD have occurred among both men and women. However, the year-on-year decrease in CHD has become progressively smaller over the last few years, compared with the situation when these observations began.

**Figure 1.**
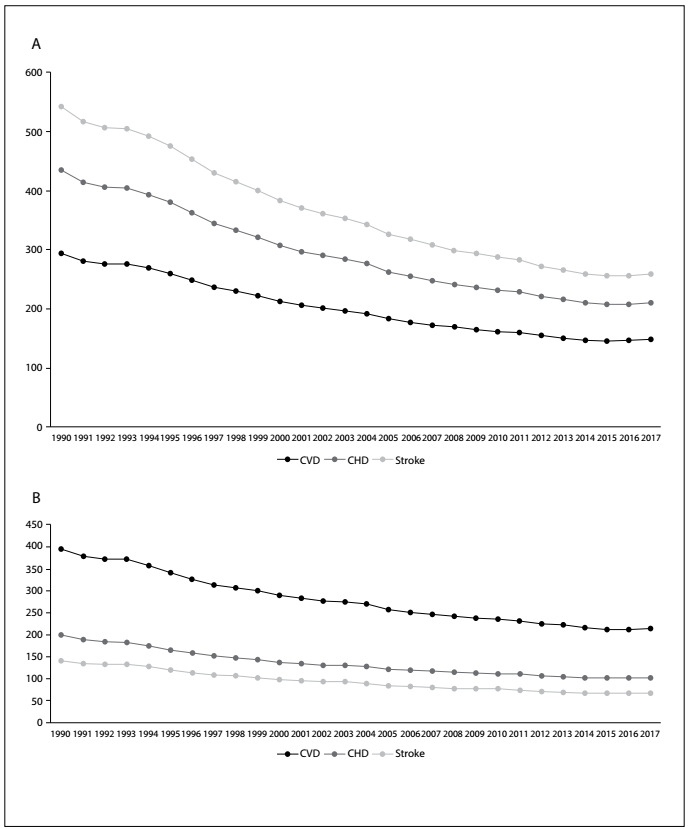
Age-adjusted death rates due to cardiovascular diseases (CVD), coronary heart disease (CHD) and stroke among men (A) and women (B) in Brazil from 1990 to 2017.^5^

**Table 1.** Annual percentage change (APC) (and 95% confidence interval) of the age-adjusted death rates among men and women in Brazil from 1990 to 2017

	Period		APC (95% confidence interval)
Men			
Cardiovascular diseases	1990	1994	-1.8 (-2.6 to 0.9)
	1994	1998	-4.0 (-5.4 to -2.7)
	1998	2007	-3.1 (-3.3 to -2.8)
	2007	2015	-2.2 (-2.6 to -1.8)
	2015	2017	0.9 (-1.8 to 3.8)
Coronary heart disease	1990	1994	-2.6 (-3.8 to -1.4)
	1994	2000	-4.4 (-5.3 to -3.6)
	2000	2009	-3.2 (-3.7 to -2.8)
	2009	2017	-1.9 (-2.3 to -1.5)
Stroke	1990	1994	-1.8 (-2.8 to 0.8)
	1994	1998	-4.7 (-6.2 to -3.2)
	1998	2008	-3.3 (-3.6 to -3.1)
	2008	2015	-2.8 (-3.3 to -2.2)
	2015	2017	1.2 (-2.0 to 4.4)
Women			
Cardiovascular diseases	1990	1994	-2.1 (-3.0 to -1.3)
	1994	1997	-4.5 (-7.1 to -1.8)
	1997	2007	-2.3 (-2.6 to -2.1)
	2007	2015	-1.9 (-2.2 to -1.5)
	2015	2017	0.4 (-2.4 to 3.2)
Coronary heart disease	1990	1994	-3.1 (-4.1 to -2.0)
	1994	1997	-5.1 (-8.2 to 1.8)
	1997	2007	-2.5 (-2.8 to -2.2)
	2007	2017	-1.6 (-1.8 to -1.3)
Stroke	1990	1994	-2.1 (-3.2 to -1.1)
	1994	1997	-5.4 (-8.4 to 2.2)
	1997	2015	-2.7 (-2.8 to -2.6)
	2015	2017	0.9 (-2.4 to 4.3)

This “almost stagnated” pattern of mortality rates for stroke and all cardiovascular diseases, but not for coronary heart disease, should serve to alert epidemiologists to the need to continue studying the determinants of cardiovascular diseases.

## References

[1] Lotufo PA, de Lolio CA (1993). Tendência da mortalidade por doença cerebrovascular no Estado de São Paulo, 1970-1989 [Mortality trends in ischemic heart disease in São Paulo State: 1970-1989]. Arq Bras Cardiol.

[2] Lotufo PA, Goulart AC, Fernandes TG, Benseñor IM (2013). A reappraisal of stroke mortality trends in Brazil (1979-2009). Int J Stroke.

[3] Brant LCC, Nascimento BR, Passos VMA (2017). Variações e diferenciais da mortalidade por doença cardiovascular no Brasil e em seus estados, em 1990 e 2015: estimativas do Estudo Carga Global de Doença [Variations and particularities in cardiovascular disease mortality in Brazil and Brazilian states in 1990 and 2015: estimates from the Global Burden of Disease]. Rev Bras Epidemiol.

[4] Wilmot KA, O’Flaherty M, Capewell S, Ford ES, Vaccarino V (2015). Coronary Heart Disease Mortality Declines in the United States From 1979 Through 2011: Evidence for Stagnation in Young Adults, Especially Women. Circulation.

[5] França EB, Passos VMA, Malta DC (2017). Cause-specific mortality for 249 causes in Brazil and states during 1990-2015: a systematic analysis for the global burden of disease study 2015. Popul Health Metr.

[6] GBD 2017 Causes of Death Collaborators (2018). Global, regional, and national age-sex-specific mortality for 282 causes of death in 195 countries and territories, 1980-2017: a systematic analysis for the Global Burden of Disease Study 2017. Lancet.

[7] Surveillance Research Program, National Cancer Institute Joinpoint Regression Program, Version 4.6.0.0 - April 2018; Statistical Methodology and Applications Branch.

